# Development of Diphenethylamines as Selective Kappa Opioid Receptor Ligands and Their Pharmacological Activities

**DOI:** 10.3390/molecules25215092

**Published:** 2020-11-02

**Authors:** Helmut Schmidhammer, Filippo Erli, Elena Guerrieri, Mariana Spetea

**Affiliations:** Department of Pharmaceutical Chemistry, Institute of Pharmacy and Center for Molecular Biosciences Innsbruck (CMBI), University of Innsbruck, Innrain 80-82, 6020 Innsbruck, Austria; filippo.erli@student.uibk.ac.at (F.E.); eleguerrieri@hotmail.com (E.G.)

**Keywords:** kappa opioid receptor, diphenethylamines, design and synthesis, structure–activity relationships, agonist, partial agonist, biased agonist, antagonist, binding affinity, selectivity

## Abstract

Among the opioid receptors, the kappa opioid receptor (KOR) has been gaining substantial attention as a promising molecular target for the treatment of numerous human disorders, including pain, pruritus, affective disorders (i.e., depression and anxiety), drug addiction, and neurological diseases (i.e., epilepsy). Particularly, the knowledge that activation of the KOR, opposite to the mu opioid receptor (MOR), does not produce euphoria or leads to respiratory depression or overdose, has stimulated the interest in discovering ligands targeting the KOR as novel pharmacotherapeutics. However, the KOR mediates the negative side effects of dysphoria/aversion, sedation, and psychotomimesis, with the therapeutic promise of biased agonism (i.e., selective activation of beneficial over deleterious signaling pathways) for designing safer KOR therapeutics without the liabilities of conventional KOR agonists. In this review, the development of new KOR ligands from the class of diphenethylamines is presented. Specifically, we describe the design strategies, synthesis, and pharmacological activities of differently substituted diphenethylamines, where structure–activity relationships have been extensively studied. Ligands with distinct profiles as potent and selective agonists, G protein-biased agonists, and selective antagonists, and their potential use as therapeutic agents (i.e., pain treatment) and research tools are described.

## 1. Introduction

Throughout human history, opioids have been used for medicinal and recreational practices to relieve pain, cough, and diarrhea, and to induce euphoria. The mood changes produced by opioids have been the basis for their abuse [1,2]. However, it was in the 20th century when major advances were made in understanding how opioids act to produce their beneficial and harmful effects. At the same time, the efficacy of opioids to treat human diseases was significantly improved by major developments in medicinal chemistry, bioinformatics, and neurosciences. Specifically, the existence of specific receptors for opioid drugs was first demonstrated on the basis of binding assays in brain preparations [3,4,5]. The multiplicity of opioid receptors was reported with different classes of opioid drugs having distinct pharmacological activities [6]. Since their first characterization, four opioid receptor types, referred to as mu (µ, MOR), delta (δ, DOR), kappa (κ, KOR), and nociceptin (NOP) receptor have been defined, and their crystal structures were obtained [7,8,9]. Opioid receptors belong to the family of seven transmembrane G protein-coupled receptors (GPCRs) and share about 60% homology in the amino acid composition [7,8,9]. Parallel to the multiple opioid receptors, endogenous opioid peptides (i.e., β-endorphins, enkephalins, dynorphins, and nociceptin/orphanin FQ) were identified in mammals [7,8]. When multiple types of opioid receptors were first proposed [6], it was clear that most of the therapeutic and side effects of opioids, clinically used as analgesics, were mediated by the MOR. These drugs included morphine and other analgesics, such as oxycodone, oxymorphone, and fentanyl [10,11,12,13]. Particularly, the misuse and overdosing with MOR agonists are an ongoing public health crisis worldwide, owing to the dramatic growth, especially in the United States of America (USA), of overdose deaths and diagnoses of opioid-use disorder associated with prescription opioids [14,15]. The challenge of researchers has always been to find innovative drugs that retain analgesic efficacy without the debilitating side effects of conventional MOR agonists, especially respiratory depression, as the primary cause of opioid-related overdose mortality.

During the past decades, the KOR has emerged as an alternative pharmacotherapeutic target; opposite to the MOR activation, the KOR does not mediate the rewarding effects or the respiratory depression, and it induces fewer gastrointestinal-related complications [7,16,17]. The endogenous kappa opioid system comprises the KOR and endogenous peptide ligands, the dynorphins, named after the Greek word dynamis (power) [18]. The KOR protein contains 380 amino acids, with seven transmembrane α-helices characteristic of the GPCRs. In comparison to other species, the human protein sequence of the KOR is 91% identical to the guinea pig and 94% identical to the mouse and rat [9,16]. The KOR/dynorphin system has a widespread distribution in the central and peripheral nervous systems (CNS and PNS) and various non-neuronal tissues of different species, including humans [19,20,21,22,23,24,25,26], consistent with its functional diversity. The endogenous kappa opioid system has key functions in numerous physiological and behavioral responses, including pain inhibition, diuresis, response to stress, reward processing, regulation of mood states, cognitive function, epileptic seizures, and sensation of itch [2,16,27,28,29,30,31].

Differential modulation of the KOR using selective ligands targeting the receptor is regarded a viable strategy for developing therapies for human disorders where the endogenous kappa opioid system plays a central role. Activation of central and peripheral KORs by agonist ligands was demonstrated to produce therapeutic effects of analgesia [32,33,34], antipruritic effects [25,35], and anticonvulsant/antiseizure effects [31,36]. Recent evidence has uncovered potential therapeutic areas for KOR antagonists, such as affective disorders and addiction-related behaviors [37,38,39,40,41,42,43,44].

Although targeting the KOR for the development of new drugs is promising, the KOR is not devoid of undesirable side effects, with receptor activation causing dysphoria, sedation, diuresis, and psychotomimetic effects in humans [33,45,46,47], and aversion, anhedonia-like, and anxiety-like effects in animals [48,49]. The contemporary concept of “functional selectivity” or “biased agonism” at the GPCR was introduced to describe the condition wherein ligands stabilize different conformations of the GPCR and can signal through parallel or independent signaling pathways mediated by G proteins and other effectors, principle among them being β-arrestin2 [50,51,52,53]. Activation of G protein-mediated pathways upon KOR activation is recognized to be responsible for the beneficial effects of analgesia and anti-itching, while β-arrestin2 recruitment and subsequent p38 phosphorylation KOR are considered to be involved in the negative side effects of dysphoria/aversion and sedation [54,55]. These findings that independent signaling mechanisms can be linked to distinct physiological effects of the KOR and can be pharmacologically separated by biased KOR ligands offer nowadays new perspectives in the discovery of KOR-targeted therapeutics with less liability for undesirable side effects. Accumulated literature on the development of KOR biased agonists was presented in recent reviews [56,57,58,59,60].

The first selective KOR ligand was ketocyclazocine (after which the kappa receptor type is named [6]), which produces analgesia in animals, as well as sedation and ataxia [33]. Over the years, a diversity of KOR ligands, as natural, naturally derived, and synthetic compounds with different scaffolds, as small molecules or peptides, with short- or long-acting pharmacokinetics, and central or peripheral site of action, were made available through chemical synthesis and evaluated as potential therapeutic agents or research tools. Several KOR agonists were evaluated in human clinical trials for the treatment of pain and pruritus, whereas KOR antagonists are under clinical development for the treatment of major depressive disorders and substance use disorders. Currently, nalfurafine is the only KOR agonist approved for clinical use as an antipruritic drug in Japan [35]. A synopsis of the literature on such developments is beyond the scope of this review; however, we recommend extended and recent reviews in the field [25,33,34,43,44,56,61,62,63,64,65,66]. Furthermore, the available crystal structures of the KOR in inactive (Protein Data Bank, PDB code 4DJH) [67] and in active conformations (PDB code 6B73) [68] and the accessibility of modern computational methods (e.g., molecular dynamics (MD) simulation) offer a unique prospect for computational drug discovery [69,70,71].

In this review, we present recent chemical developments and structure–activity relationships (SAR) for a new class of KOR ligands with a diphenethylamine scaffold. We also outline the in vitro and in vivo pharmacological activities of diphenethylamines with diverse profiles ranging from potent and selective agonists to G protein-biased agonists and selective antagonists; their potential use as therapeutics is also discussed.

## 2. Design and Synthesis of Diphenethylamines

**Figure 1:**
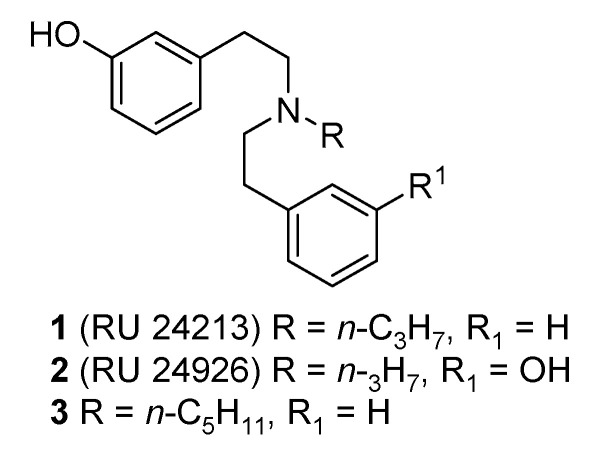
Structures of diphenethylamines **1**–**3**.

The synthesis of diphenethylamines, **1** (RU 24213) and **2** (RU 24294) (Figure 1), was described about 40 years ago [72]. These compounds were originally developed as potential anti-Parkinson’s drugs [72]. Both **1** and **2** were selective dopamine D_2_ receptor agonists at doses close to apomorphine, while exhibiting a longer duration of action [73]. Compounds **1** and **2** were also reported to display moderate affinity at the KOR acting as antagonists in in vitro binding assays [74]. The *N*-*n*-C_5_H_11_ analogue **3** (Figure 1) was tested in vivo and showed an antagonistic action against the KOR agonist U50,488 on diuresis and analgesic activity in rats [75].

**Figure 2:**
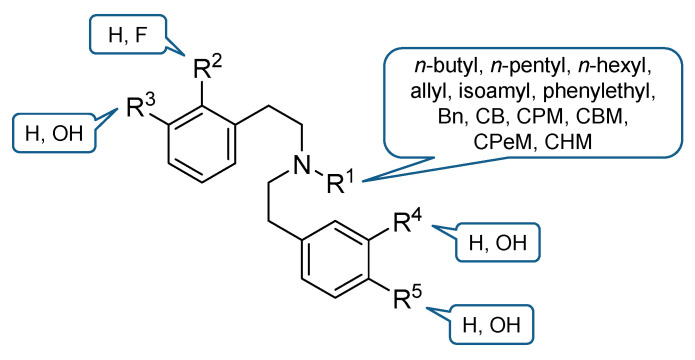
Design strategy of new KOR ligands with a diphenethylamine scaffold. Bn: benzyl, CB: cyclobutyl, CPM: cycloproplymethyl, CBM: cyclobutylmethyl, CPeM: cyclopentylmethyl, CHM: cyclohexylmethyl.

On the basis of these earlier findings, new diphenethylamine analogues with different substituents at the nitrogen and on the phenolic moieties were designed, synthesized, and biologically evaluated [76,77,78,79,80,81]. SAR studies were reported on these new structures with several diphenethylamines emerging as highly potent and selective ligands with full/partial agonist, biased agonist, and antagonist activities at the KOR (Figure 2).

### 2.1. Synthesis of 3-Monohydroxy-Substituted Diphenethylamines

**Scheme 1:**
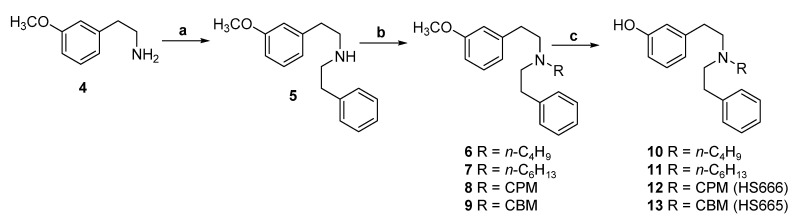
Synthetic route to diphenethylamines **10**–**13**. (**a**) Compound **4** was alkylated with phenethylamine in DMF using K_2_CO_3_ as base to give **5**; (**b**) further alkylation of **5** with the respective alkyl bromides in DMF afforded **6**–**9**; (**c**) compounds **6**–**9** were in turn transformed by ether cleavage with sodium ethanethiolate in DMF at elevated temperature into the respective phenols **10**–**13**.

The initial design strategy of new diphenethylamines as KOR ligands targeted modifications at the nitrogen with the extension of the *n*-alkyl (*n*-C_4_H_9_ and *n*-C_6_H_13_) and introduction of cycloalkylmethyl (CPM and CBM) substituents in 3-monohydroxy-substituted diphenethylamines [76]. 2-(3-Methoxyphenyl)-*N*-phenethylethaneamine (**5**) was readily available from 2-(3-methoxyphenyl) ethaneamine (**4**) by alkylation with phenethyl bromide [74]. *N*-Alkylation of **5** using the respective alkyl bromides occurred, leading to compounds **6**–**9**, which were in turn transformed by ether cleavage with sodium ethanethiolate in *N,N*-dimethylformalide (DMF) at elevated temperature into the respective phenols **10**–**13** (Scheme 1).

**Scheme 2:**
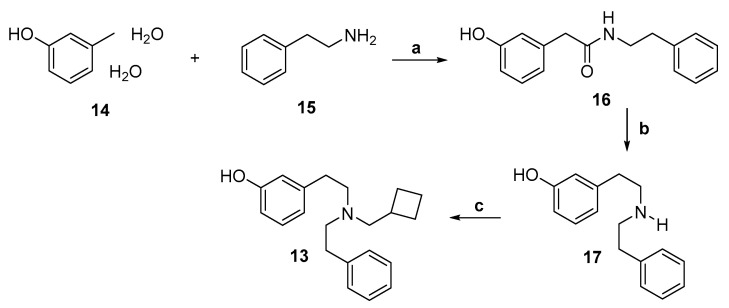
Alternative synthesis of HS665 (**13**) via acetamide **16**. (**a**) EDCl, HOAt, CH_2_Cl_2_, room temperature (r.t.); (**b**) 1 M BH_3_·tetrahydrofurane (THF), THF, reflux; (**c**) cyclobutylmethyl bromide, NaHCO_3_, CH_3_CN, reflux to afford HS665 (**13**).

The synthesis of HS665 (**13**) was optimized employing an alternative route (Scheme 2). 3-Hydroxyphenylacetic acid (**14**) was reacted with 2-phenethylamine (**15**) to afford amide **16**. Boron hydrate (BH_3_) reduction yielded amine **17**, which was *N*-alkylated with cyclobutylmethyl bromide to give **13** (HS665) with a higher yield (21%) than in the original procedure (17%) [76].

**Scheme 3:**
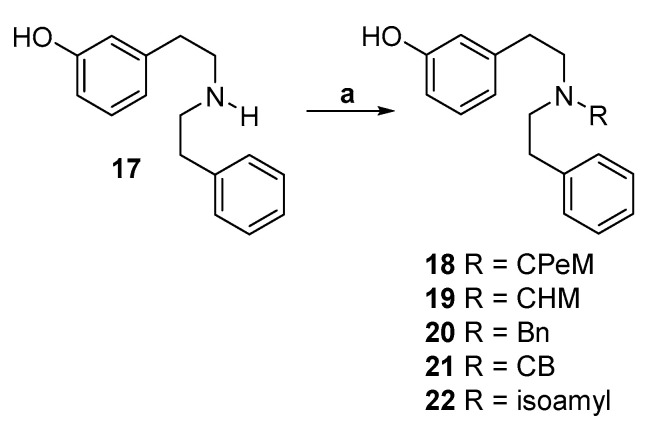
Synthetic route to diphenethylamines **18**–**22**. (**a**) Compounds **18**–**22** were prepared from **17** by *N*-alkylation with the respective alkyl bromide or cyclobutyl tosylate in the presence of NaHCO_3_ in CH_3_CN.

**Scheme 4:**
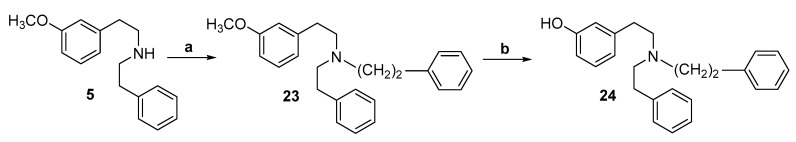
Synthetic route to diphenethylamine **24**. (**a**) Phenethyl bromide, K_2_CO_3_, DMF; (**b**) sodium ethanethiolate, DMF at elevated temperature.

The design of further 3-monohydroxy-substituted diphenethylamines as KOR ligands was based on HS666 (**12**) and HS665 (**13**) as lead molecules. The *N*-CPM and *N*-CBM groups in HS666 (**12**) and HS665 (**13**), respectively, were substituted by aliphatic and arylakyl groups of different sizes and lengths [77,78]. The synthesis of 3-monohydroxy-substituted diphenethylamines **18**–**22** started from the amine **17**, which was *N*-alkylated with cyclopentylmethyl bromide, cyclohexylmethyl bromide, benzyl bromide, isoamyl bromide, and cyclobutyl tosylate to yield the respective compounds (Scheme 3). Using this synthetical route, ether cleavage with sodium ethanethiolate was avoided. The *N*-phenylethyl-substituted diphenethylamine, **24**, was also prepared, where **5** was *N*-alkylated with phenethyl bromide to afford compound **23**, which was demethylated by ether cleavage with sodium ethanethiolate in DMF to yield phenol **24** (Scheme 4).

### 2.2. Synthesis of 4-Monohydroxy-Substituted Diphenethylamines

**Scheme 5:**
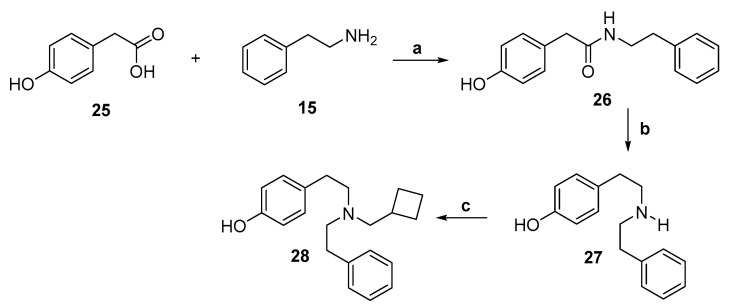
Synthetic route to diphenethylamine **28.** (**a**) EDCl, HOAt, CH_2_Cl_2_, r.t.; (**b**) 1 M BH_3_·THF, THF, reflux; (**c**) cyclobutylmethyl bromide, NaHCO_3_, CH_3_CN, reflux to afford compound **28**.

**Scheme 6:**
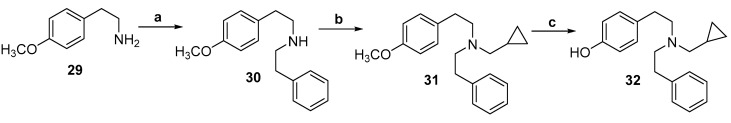
Synthetic route to diphenethylamine **32**. (**a**) Compound **29** was alkylated with phenethylamine in DMF to give **30**; (**b**) alkylation of **30** with cyclopropylmethyl bromide in DMF afforded **31**; (**c**) compound **31** was in turn transformed by ether cleavage with sodium ethanethiolate in DMF into **32**.

The next design strategy evaluated the consequence of switching the hydroxyl group from position 3 to 4 in HS666 (**12**) and HS665 (**13**) on the KOR activity [77]. The 4-monohydroxy diphenethylamines **28** and **32** were synthesized (Scheme 5 and Scheme 6). Amide **26** was prepared from 4-hydroxyphenylacetic acid (**25**), which was treated with phenethylamine (**15**) in CH_2_Cl_2_ in the presence of EDCl and HOAt. BH_3_ reduction yielded amine **27** that was *N*-alkylated with cyclobutylmethyl bromide to give **28** (Scheme 5). Alkylation of 2-(4-methoxyphenyl)ethaneamine (**29**) with phenethyl bromide afforded 2-(4-methoxyphenyl)-*N*-phenethylethaneamine (**30**), which was *N*-alkylated with cyclopropylmethyl bromide leading to **31**. Compound **31** was treated at elevated temperature with ethanethiolate in DMF to yield **32** (Scheme 6).

### 2.3. Synthesis of 3,3′-Dihydroxy-Substituted Diphenethylamines

**Scheme 7:**
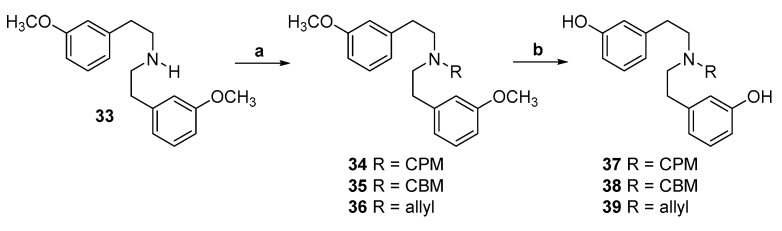
Synthetic route to diphenethylamines **37**–**39**. (**a**) Compound **33** [72] was alkylated with the respective alkyl or allyl bromide, K_2_CO_3_, DMF, N_2_, 80 °C; (**b**) sodium ethanethiolate, DMF, N_2_, 130 °C.

**Scheme 8:**
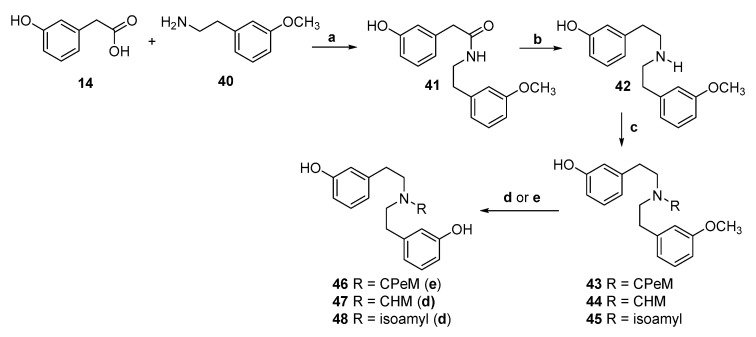
Synthetic route to diphenethylamines **46**–**48**. (**a**) EDCl and HOAt in CH_2_Cl_2_, N_2_, r.t.; (**b**) BH_3_·THF 1 M in THF, N_2_, reflux; (**c**) respective alkyl bromide, NaHCO_3_, CH_3_CN, N_2_, reflux; (**d**) sodium ethanethiolate, DMF, N_2_, 130 °C; (**e**) BBr_3_ 1 M CH_2_Cl_2_ solution in CH_2_Cl_2_, −15 °C [82].

RU 24294 (**2**) is the analogue of RU 24213 (**1**) having two hydroxyl groups at positions 3 and 3′ of the two phenyl rings (Figure 1). This compound was also found to be a selective KOR antagonist in in vitro binding assays [74]. The subsequent design strategy targeted the effect of an introduction of an additional hydroxyl group in position 3′ on the KOR activity [78]. Several 3,3′-dihydroxy diphenethylamines substituted at the nitrogen with CPM (**37**), CBM (**38**), allyl (**39**), cyclopentylmethyl (CPeM) (**46**), cyclohexylmethyl (CHM) (**47**), and isoamyl (**48**) were synthesized (Scheme 7 and Scheme 8). The synthesis of the 3,3′-dihydroxy derivatives **37**–**39** started from *N*-(3-methoxyphenethyl)-2-(3-methoxyphenyl)ethaneamine (**33**) [72], which was alkylated with the respective alkyl bromides or allyl bromide to afford **34**–**36**. Ether cleavage with sodium ethanethiolate in DMF resulted in 3,3′-dihydroxy-substituted diphenethylamines **37**–**39** (Scheme 7). 3-Hydroxyphenylacetic acid (**14**) was reacted with 3-methoxyphenylethylamine (**40**) to provide amide **41**. BH_3_ reduction in THF gave amine **42**, which was *N*-alkylated with the respective cyclopentylmethyl, cyclohexylmethyl, and isoamyl bromide to give **43**–**45**. Ether cleavage afforded **46**–**48** (Scheme 8).

### 2.4. Synthesis of 3,4′-Dihydroxy-Substituted Diphenethylamines

**Scheme 9:**
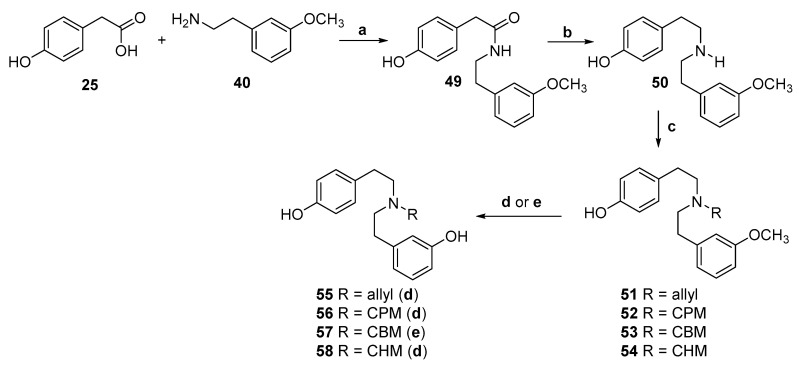
Synthetic route to diphenethylamines **55**–**58**. (**a**) EDCl and HOAt in CH_2_Cl_2_, N_2_, r.t.; (**b**) BH_3_·THF 1 M in THF, N_2_, reflux; (**c**) respective alkyl or allyl bromide, NaHCO_3_, CH_3_CN, N_2_, reflux; (**d**) sodium ethanethiolate, DMF, N_2_, 130 °C; (**e**) BBr_3_ 1 M CH_2_Cl_2_ solution in CH_2_Cl_2_, −15 °C.

Diphenethylamines with 3,4′-dihydroxy groups were designed to explore the influence of the orientation of the additional hydroxyl group on the KOR activity [78]. 3,4′-Dihydroxy diphenethylamines substituted with *N*-allyl (**55**), *N*-CPM (**56**), *N*-CBM (**57**), and *N*-CHM (**58**) were synthesized (Scheme 9). Treatment of 4-hydroxyphenylacetic acid (**25**) with 3-methoxyphenylethylamine (**40**) in the presence of EDCl and HOAt provided **49**. BH_3_ reduction of **49** in THF gave amine **50,** which was alkylated with the respective allyl or alkyl bromides **51**–**54**. Ether cleavage afforded diphenetylamines **55**–**58** (Scheme 9).

### 2.5. Synthesis of 2-Fluoro-Substituted Diphenethylamines

**Scheme 10:**
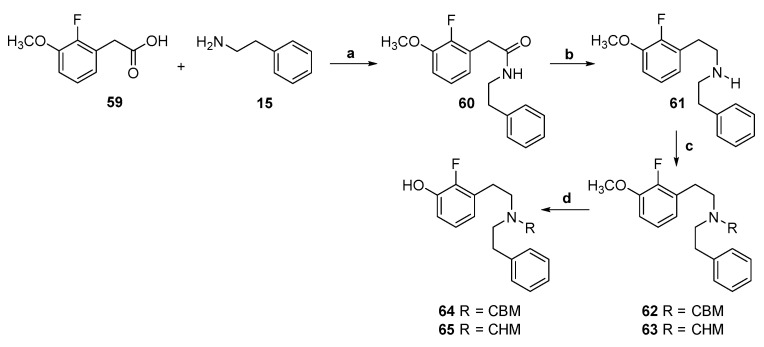
Synthetic route to diphenethylamines **64** and **65**. (**a**) EDCl and HOAt in CH_2_Cl_2_, N_2_, r.t.; (**b**) BH_3_·THF 1 M in THF, N_2_, reflux; (**c**) respective alkyl bromide, NaHCO_3_, CH_3_CN, N_2_, reflux; (**d**) BBr_3_ 1 M CH_2_Cl_2_ solution in CH_2_Cl_2_, −15 °C.

**Scheme 11:**
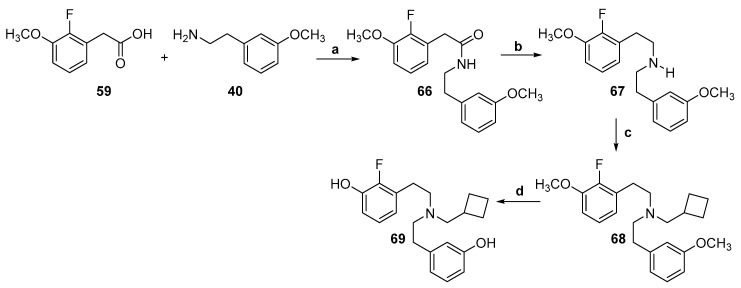
Synthetic route to diphenethylamine **69**. (**a**) EDCl and HOAt in CH_2_Cl_2_, N_2_, rt; (**b**) BH_3_·THF 1 M in THF, N_2_, reflux; (**c**) cyclobutylmethyl bromide, NaHCO_3_, CH_3_CN, N_2_, reflux; (**d**) BBr_3_ 1 M CH_2_Cl_2_ solution in CH_2_Cl_2_, −15 °C.

**Scheme 12:**
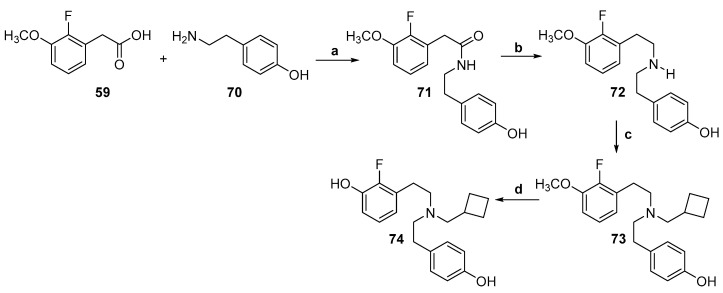
Synthetic route to diphenethylamine **74**. (**a**) EDCl and HOAt in CH_2_Cl_2_, N_2_, r.t.; (**b**) BH_3_·THF 1 M in THF, N_2_, reflux; (**c**) cyclobutylmethyl bromide, NaHCO_3_, CH_3_CN, N_2_, reflux; (**d**) BBr_3_ 1 M CH_2_Cl_2_ solution in CH_2_Cl_2_, −15 °C.

The high strength and large dipole moment of the C–F bond, along with the strong electronegativity, small size, and modest lipophilicity of fluorine, all subtend versatility in the drug design [83]. Different 2-fluoro-substituted derivatives were designed in order to assess the effect of the presence of fluorine in position 2 in diphenethylamines on the KOR activity and physicochemical properties [78]. The inclusion of a 2-fluoro substitution initially targeted the 3-monohydroxy *N*-CBM-substituted HS665 (**13**) resulting in derivative **64** (Scheme 10). Other 2-fluoro-substituted diphenethylamines were prepared including the 3-monohydroxy *N*-CHM-substituted **65** (Scheme 10), the 3,3′-dihydroxy *N*-CBM-substituted **69** (Scheme 11), and the 3,4′-dihydroxy *N*-CBM-substituted **74** (Scheme 12). It was also expected that the blood–brain barrier (BBB) penetration may be slightly restricted due to the fluoro substituent in the proximity to the 3-hydroxyl group, which results in a lower calculated pKa value of the 3-OH group of **64** in comparison to the calculated pKa value of the 3-OH group of **13** (*c*pKa = 9.77 for **13** vs. 8.35 for **64**, MarvinSketch 17.10, ChemAxon) [78]. According to the calculated partition coefficients (*c*logP) and distribution coefficients at pH 7.4 (*c*logD_7.4_) (MarvinSketch 17.10, Chem Axon), fluorinated compounds **64**, **65**, **69**, and **74** had similar values to analogues HS665 (**13**), **19**, **38**, and **57**, respectively, and a good capability to pass the BBB [78]. 2-Fluoro-3-methoxyphenylacetic acid (**59**) was treated with phenethylamine (**15**) in CH_2_Cl_2_ in the presence EDCl and HOAt to afford amide **60**. BH_3_ reduction yielded amine **61**, which was *N*-alkylated with the respective cyclobutylmethyl and cyclohexylmethyl bromide to give **62** and **63**. Ether cleavage with BBr_3_ afforded **64** and **65** (Scheme 10).

2-Fluoro-3-methoxyphenylacetic acid (**59**) was reacted with 3-methoxyphenethylamine (**40**) to provide amide **66**. BH_3_ reduction in THF gave amine **67**, which was *N*-alkylated with cyclobutylmethyl bromide to provide **68**. Ether cleavage with BBr_3_ afforded **69** (Scheme 11). Amide **71** was prepared from 2-fluoro-3-methoxyphenylacetic acid (**59**), which was treated with 3-methoxyphenethylamine (**70**) in CH_2_Cl_2_ in the presence of EDCl and HOAt. BH_3_ reduction yielded amine **72**, which was *N*-alkylated with cyclobutylmethyl bromide to give **73**. Ether cleavage with BBr_3_ afforded **74** (Scheme 12).

### 2.6. Synthesis of an Aromatic Unsubstituted Diphenethylamine

**Scheme 13:**
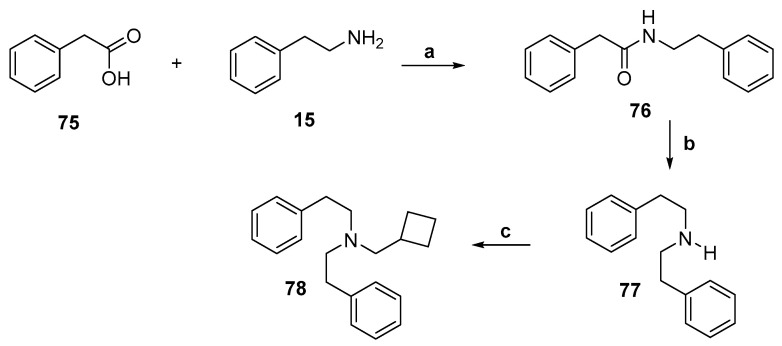
Synthetic route to diphenethylamine **78**. (**a**) EDCl and HOAt in CH_2_Cl_2_, N_2_, r.t.; (**b**) BH_3_·THF 1 M in THF, N_2_, reflux; (**c**) cyclobutylmethyl bromide, NaHCO_3_, CH_3_CN, N_2_, reflux.

To extend the understanding of the role of phenolic functions on the KOR activity, the aromatic unsubstituted diphenethylamine **78** was synthesized [78]. Amide **76** was prepared from phenylacetic acid (**75**), which was treated with 2-diphenethylamine (**15**) in CH_2_Cl_2_ in the presence EDCl and HOAt. BH_3_ reduction yielded amine **77**, which was *N*-alkylated with cyclobutylmethyl bromide to give **78** (Scheme 13).

### 2.7. Synthesis of [^3^H]HS665

**Scheme 14:**
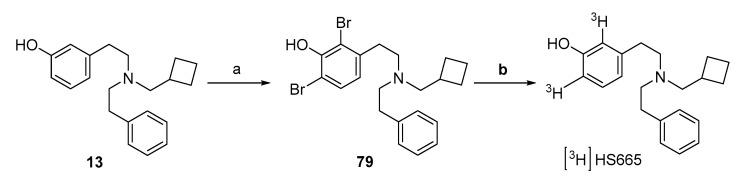
Synthetic route to [^3^H]HS665. (**a**) DIPA, NBS, CH_2_Cl_2_, r.t.; (**b**) Triethylamine (TEA), PdO/BaSO_4_ catalyst, tritium gas, r.t. HS665 (**13**) was first brominated using NBA and DIPA in CH_2_Cl_2_ to afford the dibrominated analogue **79**. A mixture of **79**, DMF, TEA, and PdO/BaSO_4_ catalyst was reacted with tritium gas at r.t. The crude product was obtained with 216 mCi in ethanol. This product was purified by RP-HPLC to afford [^3^H]HS665 with a radioactive purity of ≥95% and with a specific activity of 30.65 Ci/mmol.

In addition to their potential as therapeutics for the treatment of human disorders where the KOR has a key function, KOR ligands with a diphenethylamine scaffold may be of significant value as research tools in investigating KOR pharmacology in vitro and in vivo. Radioligands are essential tools in the GPCR research, and the field of opioid drug discovery has benefited significantly from the structural and functional insights afforded by radiolabeled ligands [84,85]. Typically, tritium labeling of small molecules and opioid peptides has a long tradition and is a well-established method to obtain labeled compounds [84,85]. Precursors for tritiation can be prepared by a number of chemical modifications, resulting in derivatives that can be reduced by tritium to obtain tritium-labeled molecules. One of the most common chemical modifications is bromination. Tritium-labeled HS665 (**13**), [^3^H]HS665, was prepared using this strategy [86]. The 2,4-dibrominated compound (**79**) was prepared from HS665 (**13**) using *N*-bromosuccinimide (NBS) and diisoproplyamine (DIPA) in CH_2_Cl_2_, and dehalotritiation of **79** was performed (Scheme 14) to yield [^3^H]HS665 with a specific activity of 30.65 Ci/mmol. [^3^H]HS665 specifically labeled the recombinant and neuronal KOR [86], and was employed as research probe for assessing in vitro KOR activity of new ligands [87,88,89,90,91,92].

### 2.8. Synthesis of Structurally Related Diphenethylamines

**Figure 3:**
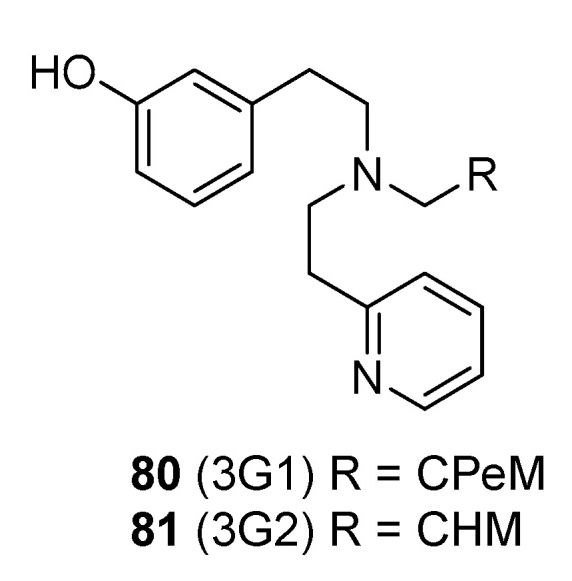
Structures of 2-pyridyl analogues **80** and **81**.

Two structurally related diphenethylamines included the 2-pyridyl analogues 3G1 (**80**) and 3G2 (**81**) (Figure 3) [80], as derivatives of the reported 3-monohydroxy, *N*-CPeM-substituted **18** and 3-monohydroxy, *N*-CHM-substituted **19** (Scheme 3) [78]. The synthesis of **80** and **81** was performed by a contract research organization (WuXi Apptech, Shanghai, China) using methods reported by the Schmidhammer and Spetea group [78], and they were described as ligands with KOR agonist activity [80].

## 3. Pharmacological Activities of Diphenethylamines at the KOR

### 3.1. Agonists and Partial Agonists

The first strategy used for designing KOR ligands with a diphenethylamine scaffold targeted the extension of the *n*-alkyl substituent at the nitrogen in the 3-monohydroxy-substituted RU 24213 (**1**) and **3** (Figure 1), specifically, substitution of the *n*-C_3_H_7_ group in **1** with an *n*-C_4_H_9_ group (**10**) and *n*-C_5_H_11_ group in **3** with an *n*-C_6_H_13_ group (**11**) (Table 1) [76]. In vitro binding studies demonstrated that replacing the *N*-*n*-C_3_H_7_ group with an *N*-*n*-C_4_H_9_ substituent (**1** vs. **10**) resulted in comparable affinity and potency at the KOR, albeit with a lower selectivity of **10** for the human KOR expressed in Chinese hamster ovary (CHO-hKOR) cells ((Table 1). Lengthening of the *N*-substituent in the *n*-C_5_H_11_ analogue **3** by one methylene group resulting in the *n*-C_6_H_13_ analogue **11** produced an additional reduction in both affinity and selectivity for the KOR. 3-Monohydroxy-substituted **1** and **3** were reported to display moderate affinity and selectivity at the KOR in the rat brain [74,75], whereas a higher KOR affinity was measured to the recombinant human receptor (Table 1) [76]. In vitro assays using rat brain membranes established diphenethylamines **1** and **3** as KOR antagonists on the basis of the decreased ligand affinity in the presence of NaCl/guanosine triphosphate [74,75]. In the guanosine-5’-O(3-[^35^S]thio)-triphosphate ([^35S^]GTPγS) binding studies using CHO-hKOR cell membranes, **1** and **3** had moderate KOR potencies and low efficacies, with a KOR partial agonist profile [76]. Extension of the *N*-*n*-alkyl substituent in 3-monohydroxy-substituted **1** and **3** conserved the KOR partial agonism character with a moderate potency for **10** and **11**, respectively (Table 1).

The subsequent design strategy evaluated the character of the *N*-substituent in 3-monohydroxy-substituted diphenethylamines (alkyl vs. cycloalkylmethyl vs. arylakyl) on KOR activities (Table 1) [76,78,81]. The *N*-CPM substituent in derivative **12** (HS666) and an *N*-CBM group in analogue **13** (HS665) were found to be more favorable for ligand binding at the KOR than the *N*-*n*-alkyl groups in **1**, **3**, **10**, and **11**, by increasing affinity and selectivity at the KOR, as well as showing better KOR agonist potency and efficacy (Table 1). HS665 (**13**) was the first 3-monohydroxy-substituted diphenethylamine reported with a KOR full agonist activity [76].

The available structures of the human KOR [67,68], together with efficient computational methods (i.e., molecular docking and molecular dynamics (MD) simulations), currently enable investigating ligand–receptor interactions, revealing structural features that promote binding and selectivity, and developing structure–affinity and structure–function relationships [69,70]. Molecular docking studies using the crystal structure of the human KOR (PDB code 4DJH) [67] and an active-like structure of the KOR attained by MD simulations explored binding modes of the 3-monohydroxy-substituted diphenethylamines, **1, 3**, and **10**–**13** at the KOR [77]. In silico studies established that the size of the *N*-substituent hosted by the hydrophobic pocket formed by the residues Val108, Ile316, and Tyr320 influenced ligand binding and selectivity to the receptor, with the *N*-CPM group in **12** (HS666) and *N*-CBM substituent in **13** (HS665) having the optimal size. The hydrogen bond formed by the phenolic 3-hydroxyl group of *N*-CBM-substituted HS665 (**13**) with His291 was crucial for binding affinity and agonist activity at the KOR [77]. Using mutant KOR models, where Val108 was virtually mutated into Ala, the corresponding residue in MOR and DOR, confirmed the experimentally demonstrated KOR selectivity of HS665 (**13**). Docking of HS665 (**13**) to the mutant receptor showed the loss of the crucial hydrogen bond of the phenolic group with His291 [77]. Molecular modeling studies also established that the *n*-alkyl size limit of the *N*-substituent is five carbon atoms, as KOR affinities of the *N*-substituted *n*-C_3_H_7_, *n*-C_4_H_9_, and *n*-C_5_H_11_ analogues were in the same range, whereas the presence of an *n*-C_6_H_13_ chain at the nitrogen caused a larger reduction in the binding affinity (Table 1) [77].

The design of new 3-monohydroxy-substituted diphenethylamines was based on HS666 (**12**) and HS665 (**13**) as leads, where the *N*-CPM substituent in HS666 (**12**) and *N*-CBM substituent in HS665 (**13**) were exchanged by aliphatic and arylakyl groups of different sizes and lengths [77,78]. Interesting SAR observations were reported in this series (compounds **18**–**22**) with regard to the KOR binding affinity, selectivity, and functional activity (Table 1). The presence of bulkier *N*-substituents, such as *N*-CPeM (**18**) and *N*-CHM (**19**) groups, resulted in the largest increase in the KOR affinity, in the picomolar range, and excellent KOR selectivity (Table 1) [78]. Introduction of an *N*-benzyl group resulted in very high affinity and selectivity at the KOR of analogue **20**, albeit less prominent than derivatives with *N*-CBM (**13**), *N*-CPeM (**18**), and *N*-CHM (**19**) substitutions. In the [^35S^]GTPγS binding assay using CHO-hKOR cell membranes, the 3-monohydroxy-substituted diphenethylamines **18** and **20** were potent and full agonist at the KOR, while derivative **19** had a profile of a potent KOR partial agonist (Table 1) [78]. Introduction of *N*-cyclobutyl (**21**) and *N*-isoamyl (**22**) substitutions was reported to produce a further decrease in the KOR affinity and selectivity and to behave as KOR partial agonists. It was established that *N*-CPeM and *N*-CHM substitutions are highly favorable in terms of interaction with the KOR [78].

Shifting the position of the phenolic hydroxyl group from position 3 to 4 in HS665 (**13**) significantly decreased affinity and selectivity at the KOR of **28** (Table 1) [77]. Differential functional activity at the KOR regarding the G protein activation was also observed after switching the hydroxyl group, with **13** as a highly potent and full KOR agonist and the 4-hydroxy modification in **28** resulting in a low potency and efficacy KOR partial agonist.

Within the 3,3′-dihydroxy diphenethylamine series (compounds **37**–**39** and **46**–**48**, Table 1), *N*-CBM (**38**), *N*-CPeM (**46**), and *N*-CHM (**47**) groups resulted in derivatives with very high KOR affinity and selectivity [78]. An additional hydroxyl group at position 3′ in *N*-CPM-substituted HS666 (**12**) and *N*-CBM-substituted HS665 (**13**) did not change the affinity and selectivity at the KOR of the resulting analogues **37** and **38**, respectively, but changed the full agonist profile of **13** into a potent KOR partial agonist **38**, with no change in the KOR partial agonism of **37** vs. **12**. Similar observation of the lack of alterations in the KOR activity was reported for the 3,3′-dihydroxy *N*-isoamyl-substituted **48** and its counterpart **22** (Table 1). The 3,3′-dihydroxy, *N*-CHM-substituted **47** had decreased KOR affinity, selectivity, and potency compared to its analogue **19**, with a shift from a partial agonist of **19** to a full agonist profile for **47**. A more significant reduction in affinity, selectivity, and potency at the KOR was found for the 3,3′-dihydroxy, *N*-CPeM derivative **46** compared to its analogue **18**, with both compounds being full agonists at the KOR. It was reported that an *N*-allyl substitution (**39**) was least favorable for the interaction with the KOR within the series of 3,3′-dihydroxy diphenethylamines (Table 1) [78].

Further SAR studies described the consequence of shifting the 3′-hydroxyl group to position 4′ with a decrease in both KOR binding affinity and selectivity for *N*-allyl-substituted **55**, *N*-CBM-substituted **57**, and *N*-CHM-substituted **58** when compared to their 3,3′-dihydroxy analogues **39**, **38**, and **47**, respectively (Table 1) [78]. In vitro functional activity studies indicated that the 3′-OH to 4′-OH shift did not change the KOR partial agonist (**39** vs. **55** and **38** vs. **57**) or full agonist activity (**47** vs. **58**).

Within the series of 2-fluorinated diphenethylamines, the *N*-CBM-substituted **64** and *N*-CHM-substituted **65** with a single 3-hydroxyl group were reported having very high affinities at the KOR, in the picomolar range, and an extraordinary KOR selectivity (Table 1) [78]. Compound **65** was the most selective KOR ligand in the series and a very potent full agonist. An *N*-CBM substitution together with a 2-fluoro substituent (**64**) significantly improved both KOR affinity and selectivity in comparison to HS665 (**13**), while it converted a full agonist (**13**) into a potent KOR partial agonist (**64**). A substantial increase in the KOR selectivity was reported for the 2-fluorinated *N*-CHM-substituted **65** compared to analogue **19**, with both compounds showing very good KOR affinity (K_i_ values of 0.061 nM for **19** and 0.040 nM for **65**), whereas **65** was also a potent full agonist. Furthermoreaddition of a 2-fluoro substituent into the 3,3′-dihydroxy, *N*-CBM derivative **38** increased binding affinity and potency at the KOR to **69**, together with an increase in the KOR selectivity for **69**, without changing the KOR partial agonism (Table 1). Whereas the 3,4′-dihydroxy, *N*-CBM-substituted **74** had comparable affinity to **14** at the KOR, the presence of a 2-fluoro substituent in **74** improved KOR selectivity and left the KOR partial agonist profile unaffected. According to these SAR observations, a fluorine substitution at position 2 in this class of diphenethylamines appears as highly advantageous with regard to the KOR activity profile [78].

The role of phenolic functions in diphenethylamines related to the KOR activity was also demonstrated, where the aromatic unsubstituted analogue **76** was synthesized and reported as having the lowest binding affinity and selectivity at the KOR within the series, showing that the presence of a 3-hydroxyl group is required for the interaction with the KOR in vitro (Table 1) [78].

Two structurally related diphenethylamines to the series presented in Table 1, the *N*-CPeM-substituted **80** and *N*-CHM-substituted **81** were reported (Figure 3, Table 2) [80]. Both compounds **80** and **81** were described as KOR full agonists in the [^35^S]GTPγS binding assay using membranes for U2OS cell stably expressing the human KOR (U2OS-hKOR) [80]. However, they had reduced agonist potencies than *N*-CPeM and *N*-CHM analogues **18** and **19**, respectively, indicating that a pyridine ring in **80** and **81** is less favorable than a phenyl ring in **18** and **19** (Table 1 and Table 2). Binding affinities and selectivities at the KOR of **80** and **81** are yet to be reported.

### 3.2. Biased Agonists

As outlined in the introduction, in addition to the G protein-mediated signaling, another important signaling event following agonist stimulation of the KOR activation is β-arrestin2 recruitment, with increasing evidence that this signaling pathway mediates the negative side effects (i.e., dysphoria/aversion, sedation, motor incoordination) associated with receptor activation. Therefore, the concept of biased agonism at the KOR has gained significance to drug discovery, with diverse chemical approaches being evaluated toward the design of G protein-biased KOR agonists as effective and safer therapeutics (for reviews, see [56,57,58,59,60]).

In vitro functional studies described diphenethylamines **10**, **12**, **13**, and **18**, as well as structurally related **80** and **81**, as G protein-biased KOR agonists with different degrees of bias (Table 2) [79,80,93]. Studies compared ligand potency and efficacy through two functional assays measuring G protein activation (the [^35^S]GTPγS binding assay) and β-arrestin2 recruitment (PathHunter β-arrestin2 assay) at the human KOR (Table 2). The *N*-*n*-C_4_H_9_ substituted **10** was found to be a KOR agonist in the [^35^S]GTPγS binding assay, without inducing any stimulation of β-arrestin2 recruitment [79]. The *N*-CPM-substituted HS666 (**12**) was reported as a KOR partial agonist in the G protein coupling assay with minor (E_max_ of 24% of the reference KOR agonist U69,593) [93] to no measurable β-arrestin2 recruitment [80]. The *N*-CBM- and the *N*-CPeM-substituted HS665 (**13**) and **18**, respectively, showed weak partial agonism for the KOR-induced β-arrestin2 recruitment, while they were very potent and fully efficacious in promoting KOR-dependent G protein activation [79,93]. A similar profile was reported for the two structurally related diphenethylamines **80** and **81** (Table 2).

### 3.3. Antinociceptive and Behavioral Effects of Agonists from the Class of Diphenethylamines

Pharmacological in vivo studies on KOR agonists from the class of diphenethylamines reported on their antinociceptive activities in mouse models of acute thermal nociception (warm-water tail-withdrawal assay) [93] and visceral pain ( acetic acid-induced writhing test) [76,78]. Evaluation of the antinociceptive effects showed that the *N*-CPM-substituted HS666 (**12**) and *N*-CBM-substituted HS665 (**13**) produced dose-dependent effects in the warm-water tail-withdrawal assay mice after central, intracerebroventricular (i.c.v.) administration (50% Effective dose, ED_50_ = 6.02 nmol for **12**, and 3.74 nmol for **13**, vs. ED_50_ = 7.21 nmol for U50,488) [93]. In the same study [93], no antinociception was detected in KOR-knockout (KO) mice, indicating that the effects of compounds **12** and **13** were KOR-specific. The first diphenethylamine reported as a potent antinociception agent following subcutaneous (s.c.) administration was the KOR full agonist HS665 (**13**) [76]. In the writhing test, HS665 (**13**) was equipotent to U50,488 (Table 3). In the mouse model of visceral pain, the KOR partial agonist HS666 (**13**) was also described as having antinociceptive efficacy after s.c. administration, with a potency twofold lower than that of U50,488 (Table 3) [78]. The antiwrithing effects of HS666 (**12**) and HS665 (**13**) were blocked by pretreatment with the KOR antagonist, nor-binaltorphimine (nor-BNI), which confirmed the KOR-specific effect [76,78].

Additional diphenethylamines with a KOR agonism profile were reported as effective antinociceptive agents with a KOR-mediated effect in the acetic acid-induced writhing test in mice after s.c. administration (Table 3) [78]. Interesting SAR observations were drawn from these studies regarding in vitro and in vivo agonist activities. In the series of 3-monohydoxy substituted diphenethylamines, the *N*-CPeM-substituted **18**, which was the most potent KOR agonist in vitro (Table 1), was described as the most effective in producing an antinociceptive effect (Table 3). The *N*-CHM-substituted **19**, a KOR partial agonist in vitro, was highly effective and only twofold less potent than its analogue **18**, while it was equipotent to the *N*-Bn-substituted **20 [78]**. Introduction of an *N*-isoamyl group (**22**) caused a reduction in the antinociceptive potency, albeit comparable to the potencies of the leads HS666 (**12**) and HS665 (**13**) (Table 3).

In the series of 3,3′-dihydroxy-substituted diphenethylamines, increasing the size of the cycloalkylmethyl substituent at the nitrogen did not significantly alter the in vivo agonist potencies, with the *N*-CBM- (**38**), *N*-CPeM- (**46),** and *N*-CHM-substituted (**47**) derivatives showing similar potencies in inhibiting the writhing response in mice after s.c. administration (Table 3) [78]. However, a reduction in the in vivo potency (twofold) was presented by the 3,3′-dihydroxy *N*-CPeM-substituted **46** compared to its 3-monohydroxy analogue **18**, an SAR observation that likely relates to the decreased in vitro KOR agonist potency of **46** (Table 1). This reduction in the antinociceptive potency was not observed for the *N*-CBM (**38**), *N*-CHM (**47**), and *N*-isoamyl (**48**) derivatives when compared to their 3-monohydroxy analogues HS665 (**13**), **19**, and **22**, respectively. It was also reported that switching the 3′-hydroxyl group to position 4′ resulted in a lower antinociceptive potency for the *N*-CHM-substituted **58** than its analogue **47** (ED_50_ = 0.95 mg/kg for **47** vs. ED_50_ = 1.90 mg/kg for **58**), whereas no difference was observed between the *N*-CBM derivatives **38** and **57** (ED_50_ = 1.71 mg/kg for **38** vs. ED_50_ = 1.73 mg/kg for **57**) (Table 3) [78].

Several diphenethylamines with a 2-fluoro substitution were reported to be highly efficacious in the writhing test in mice after s.c. administration (Table 3) [78]. Replacement of the *N*-CBM substituent in 2-fluorinated analogue **64** by the *N*-CHM group in **65** changed the in vitro agonist activity by converting a KOR partial agonist **64** into a full agonist **65** (Table 1), but also improved the antinociceptive activity of **65**. In mice, the 2-fluorinated, *N*-CHM-substituted **65** was more potent in the acetic acid-induced writhing test than its analogue **64** (Table 3). The 2-fluorinated analogues with a CBM substituent at the nitrogen, **69** and **74**, had comparable antinociceptive potencies to their *N*-CBM derivatives **38** and **57**, respectively, with all compounds reported as KOR partial agonists in vitro [78]. The 2-fluorinated, *N*-CBM-substituted **64** was slightly less potent than its analogue HS665 (**13**), associated with the reduced in vitro KOR agonist potency and efficacy of **64** (Table 1 and Table 3) [78].

In addition to antinociceptive activities of KOR agonists from the class of diphenethylamines, other behavioral responses in mice were reported (Table 4) [78,79,80,93]. Particularly, diphenethylamine derivatives with a G protein-biased agonists profile (Table 2) were evaluated for KOR-mediated side effects of sedation/motor dysfunction and aversive-like behavior. It is well recognized that agonists at the KOR cause sedation in humans, while, in animals, a decrease in locomotor activity can be measured (for reviews, see [16,33]). First in vivo studies on the *N*-CPM-substituted HS666 (**12**) and the *N*-CPM-substituted HS665 (**13**), given i.c.v. to mice at antinociceptive ED_90_ doses effective in the warm-water tail-withdrawal assay, reported on the absence of a significant effect on the motor performance in the rotarod test [93]. It was also reported that HS666 (**12**), HS665 (**13**), and two other 3-monohydroxy-substituted analogues, the *N*-CPeM- (**18**) and *N*-CHM-substituted (**19**), did not cause locomotor impairment at systemic s.c. doses up to fivefold of the antinociceptive ED_50_ doses in the writhing assay (Table 4) [78]. Intraperitoneal (i.p.) administration of HS665 (**13**) and **18** in a higher dose of 30 mg/kg to mice produced a significant decrease in the rotarod performance, but less than U50,488 [79,80]. These compounds have varying biased signaling toward G protein activation in vitro, with HS666 (**12**) as a -G protein-biased KOR partial agonist with an efficacy in β-arrestin2 recruitment ranging from 0–24% [80,93], and HS665 (**13**) and **18** as G protein-biased KOR full agonists with a partial agonist activity for β-arrestin2 signaling (efficacy between 30–55% for **13** and between 55–73% for **18**) [79,80,93].

In the rotarod test, the *N*-*n*-C_4_H_9_-substituted **10**, which does not measurably recruit β-arrestin2, did not cause locomotor incoordination following i.p. administration to mice at a dose of 30 mg/kg [79,80]. Diphenethylamines with a 2-fluoro substitution and an *N*-CBM group (**64**) or an *N*-CHM group (**65**) also produced no significant changes in the motor function of mice at doses equivalent to fivefold the effective antinociceptive ED_50_ dose in the writhing assay (Table 4) [78]. Structurally related diphenethylamines **80** and **81**, as partial agonists for β-arrestin2 signaling, caused a modest motor impairment at doses of 30 mg/kg, i.p., without reaching the level of deficiency produced by U50,488 in the rotarod test [80]. Until now, there are no data available on the antinociceptive potencies of diphenethylamines derivatives **10**, **80**, and **81**; therefore, it is difficult to evaluate the safety profile of these compounds. The in vivo findings related to the impact on the motor function of diphenethylamines **10**, HS666 (**12**), HS665 (**13**), **18**, **80**, and **81** shows a possible correlation between the level of biased agonism and KOR agonism-induced motor incoordination.

A major side effect related to the KOR activation is dysphoria, reported already in early human studies [45,46]. Because dysphoria cannot be directly measured in animals, the aversive response of a drug can be assessed using the conditioned place aversion (CPA) paradigm [48,49]. Conventional, unbiased KOR agonists (i.e., U50,488, U69,593, and salvinorin A) produce marked aversive effects in rodents in the CPA test [36,93,94,95,96]. The KOR-specific aversive effects were described to be linked with the recruitment of β-arrestin2 to the receptor, with G protein-biased KOR agonists expected to achieve the beneficial effect of analgesia and to be devoid of dysphoric/aversive effects.

First studies on the potential to induce KOR-specific aversive behavior in the CPA test were reported for the *N*-CPM-substituted HS666 (**12**) and the *N*-CPM-substituted HS665 (**13**) following central i.c.v. administration in mice [93]. Neither preference nor aversion was measured in mice after treatment with HS666 (**13**) up to 25-fold the antinociceptive ED_50_ doses effective in the warm-water tail-withdrawal assay. In the same study, HS665 (**13**) was found to induce aversive-like effects in mice in a dose eightfold higher than the effective antinociceptive ED_50_ dose in the tail-withdrawal assay [93]. The in vivo pharmacological profile of HS665 (**13**) appeared to be alike to that of the G protein biased-KOR agonist RB-64 [94], including antinociception effects with no motor incoordination, but with aversive-like actions in mice [93,94]. Furthermore, this is a notable profile, as HS665 (**13**) appears to activate favorably KOR-mediated G proteinactivation over β-arrestin2 signaling, albeit with higher efficacy for β-arrestin2 recruitment than HS666 (**12**) (Table 2) [93].

A recent study using high-throughput phosphoproteomics compared the phosphoproteomes of the mouse striatum after central, intracisternal (i.c.) injection of HS666 (**12**) and HS665 (**13**) and other KOR agonists, U50,488 [97], 6′-guanidinonaltrindole (6′-GNTI) [98], and RB-64 [99], given at doses effective in behavioral studies [95]. It was found that, compared to the aversive, β-arrestin2recruiting KOR agonists U50,488 [36,93,96], RB-64 [94,100], and HS665 (**12**) [93], the nonaversive, G protein-biased HS666 (**12**) [93] and 6′-GNTI [36,101,102] had a differential dynamic phosphorylation pattern of synaptic proteins and did not activate mTOR signaling [95]. This study proposed that the mammalian target of rapamycin (mTOR) signaling pathway may be involved in mediating aversion caused by KOR agonists. The KOR-specific mechanism was demonstrated by the lack of significant phosphorylation changes in the KOR-KO mouse striatum [95].

### 3.4. Antagonists

Diphenethylamines displaying antagonist activity at the KOR were reported [77,78]. The first diphenethylamine was the *N*-phenylethyl-substituted **24,** which showed no substantial agonist activity at the KOR in vitro, and antagonized U69,593-induced [^35^S]GTPγS binding with relatively low potency (Table 5) [77]. In vitro binding studies with CHO-hKOR cell membranes established that an *N*-phenylethyl group (**24**) also resulted in a decreased binding affinity at the KOR compared to other diphenethylamine derivatives and a complete loss of binding at the MOR and DOR (Table 5).

Switching the 3-hydroxyl group to position 4 in *N*-CPM-substituted **12** converted the KOR partial agonist HS666 (**12**) into a KOR antagonist (**32**) (Table 5) [77]. The 4-hydroxy, *N*-CBM-substituted **32** had higher KOR antagonist potency than the *N*-phenylethyl-substituted **24** in the [^35^S]GTPγS binding assay. The in vitro profiles of diphenethylamines **24** and **32** were supported by molecular docking studies [77] using the inactive structure of the human KOR (PDB code 4DJH) [67]. The *N*-phenylethyl group in **24** is relatively bulky to be hosted by the hydrophobic pocket formed by the residues Val108, Ile316, and Tyr320, which resulted in a different orientation of the phenolic moiety compared to the full agonist HS665 (**13**), making this compound a weak KOR antagonist. Diphenethylamine **32** with a phenolic 4-hydroxy group did not form the hydrogen bond with His291, an important residue for affinity and agonist activity at the KOR [77].

It was also reported that introduction of an additional hydroxyl group at position 4′ in HS666 (**13**) also changed the in vitro functional activity of **13**, from a KOR partial agonist to an antagonist **56** (Table 5) [78]. An interesting observation was that an additional hydroxyl group in position 3′ into *N*-CPM-substituted **32** retained the high antagonist potency at the KOR in vitro, while it also increased affinity and selectivity of analogue **56** at the KOR (Table 5). The in vivo KOR antagonist activity of 3,4′-dihydroxy, *N*-CPM derivative **56** was also demonstrated [78]. Pretreatment of mice with **56** (10 mg/kg, s.c.), 15 min before the KOR agonist U50,488, produced a complete reversal of U50,488-induced antinociception in the acetic acid-induced writhing assay. The absence of an agonist activity was further demonstrated for **56**, as it did not affect writhing pain behavior after s.c. administration to mice [78].

## 4. Summary and Conclusions

Potent and selective KOR ligands have been targeted since the discovery of multiple opioid receptor types, with increased attention in the 21st century paid to the discovery of novel ligands targeting the receptor and their potential to treat human disorders involving the kappa opioid system. This field has significantly advanced with an understanding of the function of the endogenous kappa opioid system in physiological and neuropsychiatric behaviors. Furthermore, future drug development in the KOR field is expected to significantly benefit from the available active and inactive KOR crystal structures and access to powerful computational systems and technologies.

In this review, we focused on a new class of KOR ligands with a diphenethylamine scaffold. We highlighted chemical advances in the functionalization and modification of the diphenethylamines toward the development of KOR ligands with distinct profiles, ranging from potent and selective agonists to G protein-biased agonists and selective antagonists. The first leads were HS666 (**12**) and HS665 (**13**), a selective KOR partial agonist and a full agonist, respectively [76]. The emerged SAR studies showed that KOR selectivity can be affected by simple structural modifications. The 3-hydroxyl function is required for the interaction with the KOR in vitro, and the character of the *N*-substituent plays an important role on the binding and activation of the KOR. The SAR established that an *N*-CPM substitution in HS666 (**12**) and an *N*-CBM substitution in HS665 (**13**) are more favorable for the interaction with KOR than *n*-alkyl groups causing an increase in KOR affinity and selectivity, as well as in KOR agonist potency and efficacy. Bulkier substituents at the nitrogen, such as CPeM and CHM (compounds **18** and **19**, respectively, Table 1), resulted in the largest increase (in the picomolar range) in binding affinity and excellent selectivity for the KOR. Furthermore, modification with a 2-fluoro substitution in *N*-CBM- and *N*-CHM-substituted diphenethylamines (**64** and **65**, respectively, Table 1) led to compounds with very high affinities (in the picomolar range) at the KOR and an additional increase in the KOR selectivity. These properties make such compounds valuable research tools in investigating KOR pharmacology. The 3-OH→4-OH switch resulted in reduced KOR binding. Additional hydroxyl groups at positions 3′ or 4′ had different consequences on the KOR activity, with the 3,4′-dihydroxy, *N*-CBM-substituted **58** (Table 4) as a high affinity and selective KOR ligand with in vitro and in vivo antagonism. Among the diphenethylamine derivatives, G protein-biased KOR agonists with different degrees of bias were identified.

Pain represents a primary clinical indication for KOR agonists, with the evidence that agonists at the KOR have analgesic properties with lower abuse potential than MOR agonists. Diphenethylamines with a KOR agonist profile (full, partial, or G protein-biased) were demonstrated as highly efficacious antinociceptive agents with a KOR-specific mechanism of action in mouse models of acute thermal nociception and visceral pain. Furthermore, behavioral studies established these ligands as potential antinociceptives with reduced liability for KOR-mediated adverse effects in mice (aversion, sedation/locomotor impairment) [78,93].

Whereas KOR antagonists are important research tools for studying the in vitro and in vivo KOR pharmacology, evidence on their antidepressant, anxiolytic, and antiaddictive effects support the potential therapeutic applications of KOR antagonists in the treatment of human disease states (i.e., depression, anxiety, and addiction). Selective KOR ligands from the class of diphenethylamines were reported with in vitro and in vivo antagonism, with future studies remaining to establish their therapeutic value.

In summary, a combination of target drug design, synthetical efforts, and pharmacological assessments of diphenethylamines as a class of structurally distinct, selective KOR ligands, enabled the identification of structural elements that determine the distinct activity profiles, with the prospective as candidates for future drug development for the treatment of pain and other neuropsychiatric illnesses.

## Figures and Tables

**Table 1:**
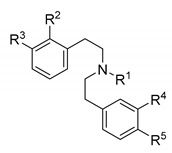
In vitro activities of differently substituted diphenethylamines as full and partial agonists at the KOR.

Ligand	R^1^, R^2^, R^3^, R^4^, R^5^	KOR Binding ^a^		KOR Activity ^b^	
		K_i_ (nM)	K_i_ ratio KOR/MOR/DOR	EC_50_ (nM)	%Stim.
**3-Monohydroxy-substituted**					
**1** (RU 24213)	*n-*C_3_H_7_, H, OH, H, H	8.13	1/73/457	49.1	21.1
**3**	*n-*C_5_H_11_, H, OH, H, H	12.6	1/26/104	86.4	36.2
**10**	*n-*C_4_H_9_, H, OH, H, H	10.9	1/38/223	46.2	45.5
**11**	*n-*C_6_H_13_, H, OH, H, H	141	1/5.6/25	647	24.0
**12** (HS666)	CPM, H, OH, H, H	5.90	1/140/>1700	35.0	53.4
**13** (HS665)	CBM, H, OH, H, H	0.49	1/1106/>20000	3.62	90.0
**18**	CPeM, H, OH, H, H	0.017	1/16118/133471	3.87	82.8
**19**	CHM, H, OH, H, H	0.061	1/8803/35066	0.23	61.9
**20**	Bn, H, OH, H, H	0.71	1/652/2623	4.65	79.5
**21**	CB, H, OH, H, H	10.3	1/65/344	46.1	50.7
**22**	isoamyl, H, OH, H, H	2.69	1/96/1020	22.1	74.7
**4-Monohydroxy-substituted**					
**28**	CBM, H, OH, H, H	36.3	1/20/59	109	43.8
**3,3′-Dihydroxy-substituted**					
**37**	CPM, H, OH, OH, H	4.62	1/137/617	20.6	51.3
**38**	CBM, H, OH, OH, H	0.38	1/605/8789	4.44	71.1
**39**	allyl, H, OH, OH, H	19.1	1/19/39	154	37.5
**46**	CPeM, H, OH, OH, H	0.31	1/1884/8952	13.7	80.4
**47**	CHM, H, OH, OH, H	0.14	1/1193/10229	17.6	91.1
**48**	isoamyl, H, OH, OH, H	2.10	1/100/699	16.6	65.6
**3,4′-Dihydroxy-substituted**					
**55**	allyl, H, OH, H, OH	43.5	1/4/25	248	29.8
**57**	CBM, H, OH, H, OH	3.43	1/5/125	22.2	76.4
**58**	CHM, H, OH, H, OH	1.85	1/126/885	22.2	84.3
**2-Fluoro-substituted**					
**64**	CBM, F, OH, H, H	0.072	1/5529/>138000	6.90	66.1
**65**	CHM, F, OH, H, H	0.040	1/21275/>250000	2.77	88.9
**69**	CBM, F, OH, OH, H	0.12	1/4642/>83000	1.49	57.5
**74**	CBM, F, OH, H, OH	3.37	1/155/389	36.7	69.1
**Unsubstituted**					
**78**	CBM, H, H, H, H	79.1	1/13/28	359	91.9

^a^ Determined in competition binding assays using membranes from CHO cells stably expressing human opioid receptors. ^b^ Determined in the [^35^S]GTPγS binding assay using CHO-hKOR cell membranes. Data from [76,77,78]. K_i_, inhibition constant; EC_50_, 50% effective concentration; %Stim, percentage stimulation relative to U69,593 (reference KOR full agonist).

**Table 2:** Comparison of in vitro potencies and efficacies of KOR agonists from the class of diphenethylamines to induce G protein activation and β-arrestin2 recruitment.

Ligand	G Protein Activation ^a^		β-Arrestin2 Recruitment ^b^		References
	EC_50_ (nM)	E_max_ (%) ^c^	EC_50_ (nM)	E_max_ (%) ^c^	
**10**	14 (GTPγS, U2OS-hKOR)	94	–^d^ (PathHunter, U2OS-hKOR-β-arrestin2)	–^d^	[79]
**12** (HS666)	35.7 (GTPγS, CHO-hKOR)	50	449 (PathHunter, U2OS-hKOR-β-arrestin2)	24	[93]
**13** (HS665)	4.987 (GTPγS, CHO-hKOR)	88	463 (PathHunter, U2OS-hKOR-β-arrestin2)	55	[93]
**18**	0.64 (GTPγS, U2OS-hKOR)	100	720 (PathHunter, U2OS-hKOR-β-arrestin2)	55	[79]
**80**	8.2 (GTPγS, U2OS-hKOR)	86.7	3956 (PathHunter, U2OShKOR-β-arrestin2)	61.2	[80]
**81**	11.8 (GTPγS, U2OS-hKOR)	81.6	2082 (PathHunter, U2OShKOR-β-arrestin2)	57.7	[80]

^a^ Determined in the [^35^S]GTPγS binding assay at the human KOR. ^b^ Determined in the PathHunter β-arrestin2 recruitment assay at the human KOR. ^c^ Percentage relative to the maximal effect of U69,593. –^d^ denotes no stimulation. For structures of compounds **10**, **12**, **13**, and **18**, refer to Table 1; for structures of compounds **80** and **81**, refer to Figure 3.

**Table 3:**
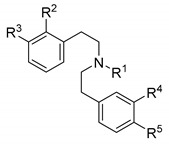
Antinociceptive potencies of differently substituted diphenethylamines in mice.

Ligand	R^1^, R^2^, R^3^, R^4^, R^5^	Antinociception ^a^ ED_50_ (mg/kg, s.c.) ^a^
U50,488	-	1.54
**12** (HS666)	CPM, H, OH, H, H	3.23
**13** (HS665)	CBM, H, OH, H, H	1.91
**18**	CPeM, H, OH, H, H	0.49
**19**	CHM, H, OH, H, H	1.01
**20**	Bn, H, OH, H, H	1.21
**22**	isoamyl, H, OH, H, H	2.78
**37**	CPM, H, OH, OH, H	4.73
**38**	CBM, H, OH, OH, H	1.71
**46**	CPeM, H, OH, OH, H	1.19
**47**	CHM, H, OH, OH, H	0.95
**48**	isoamyl, H, OH, OH, H	2.63
**57**	CBM, H, OH, H, OH	1.73
**58**	CHM, H, OH, H, OH	1.90
**64**	CBM, F, OH, H, H	2.64
**65**	CHM, F, OH, H, H	1.33
**69**	CBM, F, OH, OH, H	2.25
**74**	CBM, F, OH, H, OH	2.14

^a^ Determined in the acetic acid-induced writhing assay in mice after s.c. administration. Data from [76,78].

**Table 4:** Behavioral effects in mice of KOR agonists from the class of diphenethylamines.

Ligand	Antinociception (Test, ED_50_, Route, Strain)	Locomotor Activity (rotarod test) (Dose, Route, Strain)	Aversion (CPA) (Dose, Route, Strain)	References
**10**	n.d.	30 mg/kg, i.p., C57/BL6	n.d.	[79,80]
**12** (HS666)	tail-withdrawal, 6.02 nmol, i.c.v., C57/BL6J writhing, 3.23 mg/kg, s.c., CD1	30 nmol, i.c.v., C57/BL6J 10 and 20 mg/kg, s.c., CD1	CPA, 150 nmol, i.c.v., C57/BL6J n.d.	[93] [78]
**13** (HS665)	tail-withdrawal, 3.74 nmol, i.c.v., C57/BL6J writhing, 1.91 mg/kg, s.c., CD1	10 nmol, i.c.v., C57/BL6J 5 and 10 mg/kg, s.c., CD1 30 mg/kg, i.p., C57/BL6	CPA, 30 nmol, i.c.v. C57/BL6J n.d.	[93] [76,78] [79,80]
**18**	writhing, 0.49 mg/kg, s.c., CD1	2.5 mg/kg, s.c., CD1 30 mg/kg, i.p., C57/BL6	n.d.	[78] [79,80]
**19**	writhing, 1.01 mg/kg, s.c., CD1	5 mg/kg, s.c., CD1	n.d.	[78]
**64**	writhing, 2.64 mg/kg, s.c., CD1	15 mg/kg, s.c., CD1	n.d.	[78]
**65**	writhing, 1.33 mg/kg, s.c., CD1	7.5 mg/kg, s.c., CD1	n.d.	[78]
**80**	n.d.	30 mg/kg, i.p., C57/BL6	n.d.	[80]
**81**	n.d.	30 mg/kg, i.p., C57/BL6	n.d.	[80]

For structures of compounds **10**, **12**, **13, 18, 19**, **64**, and **65**, refer to Table 1; for structures of compounds **80** and **81**, refer to Figure 3. CPA, conditioned place aversion; i.c.v., intracerebroventricular; s.c., subcutaneous; i.p., intraperitoneal; n.d., not determined.

**Table 5:**
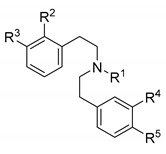
In vitro activities of differently substituted diphenethylamines as antagonists at the KOR.

Ligand	R^1^, R^2^, R^3^, R^4^, R^5^	KOR Binding ^a^		KOR Activity ^b^
		K_i_ (nM)	K_i_ Ratio KOR/MOR/DOR	K_e_ (nM)
**24**	(CH_2_)_2_Ph, H, OH, H, H	211	1/>47/>47	1311
**32**	CPM, H, H, H, OH	218	1/8/10	32.1
**56**	CPM, H, OH, H, OH	3.56	1/129/>2800	24.3

^a^ Determined in competition binding assays using membranes of CHO cells stably expressing human opioid receptors. ^b^ Determined in the [^35^S]GTPγS binding assay using CHO-hKOR cell membranes. Data from [77,78].
